# The evolution of haploid chromosome numbers in Meliponini

**DOI:** 10.1371/journal.pone.0224463

**Published:** 2019-10-24

**Authors:** Natália Martins Travenzoli, Danon Clemes Cardoso, Hugo de Azevedo Werneck, Tânia Maria Fernandes-Salomão, Mara Garcia Tavares, Denilce Meneses Lopes

**Affiliations:** 1 Laboratório de Citogenética de Insetos, Departamento de Biologia Geral, Universidade Federal de Viçosa, CEP, Viçosa, Minas Gerais, Brazil; 2 Laboratório de Genética Evolutiva e de Populações, Departamento de Biodiversidade, Evolução e Meio Ambiente, Universidade Federal de Ouro Preto, CEP, Ouro Preto, Minas Gerais, Brazil; 3 Laboratório de Biologia Molecular de Insetos, Departamento de Biologia Geral, Universidade Federal de Viçosa, CEP, Viçosa, Minas Gerais, Brazil; Facultad de Veterinaria, Universidad de Murcia, SPAIN

## Abstract

It is thought that two evolutionary mechanisms gave rise to chromosomal variation in bees: the first one points to polyploidy as the main cause of chromosomal evolution, while the second, Minimum Interaction Theory (MIT), is more frequently used to explain chromosomal changes in Meliponini and suggests that centric fission is responsible for variations in karyotype. However, differences in chromosome number between Meliponini and its sister taxa and in the karyotype patterns of the *Melipona* genus cannot be explained by MIT, suggesting that other events were involved in chromosomal evolution. Thus, we assembled cytogenetical and molecular information to reconstruct an ancestral chromosome number for Meliponini and its sister group, Bombini, and propose a hypothesis to explain the evolutionary pathways underpinning chromosomal changes in Meliponini. We hypothesize that the common ancestor shared by the Meliponini and Bombini tribes possessed a chromosome number of *n* = 18. The karyotype with *n* = 17 chromosomes was maintained in Meliponini, and variations of haploid numbers possibly originated through additional Robertsonian fissions and fusions. Thus, the low chromosome number would not be an ancestral condition, as predicted by MIT. We then conclude that Robertsonian fission and fusions are unlikely to be the cause of chromosomal rearrangements that originated the current karyotypes in Meliponini.

## Introduction

Meliponini, Bombini, Apini, and Euglossini tribes comprise those bees known as "corbiculate", and their evolutionary history has been studied through morphological, phylogenetic, and cytogenetic analyses [1–10]. Cytogenetic analyses, in particular, are an important tool for understanding the macro-scale genomic organization of different any species. These analyses comprise descriptions of chromosome number [11], [2], [9], heterochromatin distribution patterns [12], characterization of AT and CG rich regions [13], [12], localization of 18S ribosomal genes [14], [12], mapping of repetitive DNA sequences [15], and inferences of karyotype evolution [10–11].

In bees, two main hypotheses have been proposed to explain changes related to chromosome number and structure. The first indicates that changes in ploidy, through whole-genome duplication, are the main mechanism involved in chromosome evolution [16]. On the other hand, a second hypothesis, known as Minimum Interaction Theory (MIT), suggests centric fission as the main mechanism responsible for chromosome variation [11] [17–21]. According to the MIT, modifications in karyotypes that occur through centric fission in different species evolve in order to minimize the deleterious effects of chromosomal interactions. However, they generate instability in the break regions of fictional chromosomes, which then tends to be minimized by the incorporation of heterochromatin [19], [20], [21]. This would generate chromosomes presenting one heterochromatic arm and one euchromatic arm, and we would expect to find this as a common pattern in the Meliponini [22], [23], [24].

Based on this theory, the ancestor of the living species of the Meliponini tribe would present a low chromosome number, and this number would increase through changes acquired by fission and a subsequent accumulation of heterochromatin. However, when we analyzed the karyotype of other corbiculate tribes phylogenetically close to Meliponini (which vary from *n* = 08, *n* = 09, *n* = 15, *n* = 17 and *n* = 18, predominating *n* = 17), such as Bombini (*n* = 18–20), Apini (*n* = 17) and Euglossini (*n* = 20–21), we observed that they have a high chromosome number [1], [2], [8]. In addition, the heterochromatin distribution patterns of several *Melipona* species [25] seem to have arisen from events different from those proposed by MIT.

Thus, the MIT, although widely used to explain the chromosomal evolution in Meliponini, does not seem to explain the chromosomal number observed across this tribe, nor the structural variations or heterochromatic patterns observed in *Melipona*. Thus, the objective of this study was to infer the ancestral chromosome number of the Meliponini tribe and its sister group Bombini in order to evaluate potential rearrangements that lead to the evolutionary karyotypic changes. Based on this phylogenetic approach, we propose a hypothesis alternative to MIT, which may have contributed to the evolutionary processes underpinning chromosomal changes in bees.

## Material and methods

### Phylogenetic analysis and molecular dating

A total of 67 species representing 28 genera with haploid chromosome numbers described in the literature, including 50 Meliponini and 17 Bombini species, were selected to compose our dataset (Table 1). As such, we essentially reconstructed the phylogenetic hypotheses from Rasmussen and Cameron [7]. To the phylogenetic analysis, the Meliponini and Bombini tribes were considered to be the in-groups, while the outgroups were *Apis dorsata* (Fabricius, 1793), *Euglossa imperialis* (Cockerell, 1922), *Eulaema boliviensis* (Friese, 1898), and *Exaerete smaragdina* (Guérin-Méneville, 1845). Partial sequences of the following nuclear genes were used to infer the phylogenetic tree: arginine kinase (ArgK), long-wavelength rhodopsin copy 1 (Opsin), elongation factor-1α F2 (EF1-α), 28S (28S rDNA), and the mitochondrial 16S rRNA [3], [4], [7], [9]. All sequences were retrieved from Genbank and the associated accession numbers are listed in S1 Table. Sequences were aligned using MAFFT [26] and visually verified in MEGA v7.0 [27]. The nuclear genes EF-1α, Opsin, and ArgK were partitioned into exons and introns [28], [29], while 28S and 16S were considered as a single partition. The final alignments were concatenated into a single matrix in the Sequence Matrix v.1.7.8 [30]. The analyses were performed on the CIPRES Science Gateway online server [31] using Bayesian inference by the MrBayes v3.2.2 software [32] with two independent runs with four Markov Chain Monte Carlo (MCMC) in each. The mixed model [33]) was implemented for all partitions with a proportion of invariable sites and a Gamma correction. We used 50,000,000 generations of MCMC with trees sampled every 1000 generations. The convergence of the Markov chains was verified in Tracer v.1.5 [34]. Twenty-five percent of the initial trees were discarded and those that remained were used to generate the consensus tree. The trees were viewed and edited in FigTree v.1.3.1 [35].

**Table 1:** Species of bees, haploid number (*n*), karyotype formula, and references.

Species	*n*	Karyotypic formula	References
*Austroplebia australis*	18	_	Unpublished data
*Bombus* (*Bombus*) *hypocrita*	18	4A^m^+2A+1A^mi^+6M^c^+2M+2M^Ci^+1M^t^	[11]
*Bombus* (*Bombus*) *ignitus*	18	4A^m^+1A^c^+1A+1A^i^+5M^c^+3M+1M^i^+1M^C^+1M^t^	[11]
*Bombus* (*Bombus*) *terricola*	18		[2]
*Bombus* (*Cullumanobombus*) *griseocollis*	18		[2]
*Bombus* (*Cullumanobombus*) *rufocinctus*	18		[2]
*Bombus* (*Megabombus*) *diversus*	18	1A^m^+1A^Mc^+5A^c^+1A+1A^i^+1A^ci^+2M^C^+1M+1M^CC^+1M^i^+1M^Ci^+1M^t^	[11]
*Bombus* (*Pyrobombus*) *ardens*	18	3A^M^+4A^Mc^+1A^c^+1A+5M^C^+1M+2M^CC^+1M^CCT^	[11]
*Bombus* (*Pyrobombus*) *huntii*	18		[2]
*Bombus* (*Pyrobombus*) *impatiens*	18		[2]
*Bombus* (*Pyrobombus*) *perplexus*	12		[2]
*Bombus* (*Subterraneo*) *appositus*	16		[2]
*Bombus* (*Subterraneo*) *borealis*	16		[2]
*Bombus* (*Thoracobombus*) *fervidus*	18		[2]
*Bombus* (*Thoracobombus*) *pauloensis*	20		Unpublished data
*Bombus* (*Thoracobombus*) *pensylvanicus*	18		[2]
*Bombus* (*Thoracobombus*) *pseudobaicalensis*	17	1A^Mc^+1A+1A^i^+1A^Mi^+1A^Mt^+5M^C^+3M^CC^+2M^C^+2M^Ci^	[2]
*Bombus* (*Thoracobombus*) *schrencki*	17	10M^c^+1M+5M^i^+1M^Ci^	[2]
*Cephalotrigona capitata*	17	18A+16A^m^	[56]
*Dactylurina staudingeri*	17	_	[77], [78]
*Duckeola ghilianii*	15	_	[77], [78]
*Friesella schrottkyi*	17	_	[58], [79]
*Frieseomelitta trichocerata*	15	4M+16A+10A^m^	[80]
***Continuation***			
**Species**	***n***	**Karyotypic formula**	**References**
*Frieseomelitta varia*	15	4M+4A+22A^m^	[56], [58], [78], [81]
*Geotrigona mombuca*	15	2M+6A+7A^m^	[56], [58], [78]
*Lestrimelitta limao*	14	6M+6A+16A^m^	[56], [81], [82]
*Leurotrigona muelleri*	08	_	[53], [58], [77], [81]
*Leurotrigona pusilla*	15	_	[53]
*Meliplebeia becarii*	17	_	[77], [80]
*Melipona* (*Eomelipona*) *bicolor*	09	_	[54], [55], [58]
*Melipona* (*Eomelipona*) *marginata*	09	_	[54], [55], [58], [77]
*Melipona* (*Melikerria*) *fasciculata*	09	_	[55], [75], [78], [83]
*Melipona* (*Melikerria*) *quinquefasciata*	09	_	[55], [58], [75], [76], [78]
*Melipona* (*Melipona*) *favosa*	09	_	[11], [84]
*Melipona* (*Melipona*) *mandacaia*	09	_	[25]
*Melipona* (*Melipona*) *quadrifasciata*	09	_	[54], [60]
*Melipona* (*Michmelia*) *crinita*	09	_	[55]
*Melipona* (*Michmelia*) *scutellaris*	09	_	[54], [55], [58]
*Melipona* (*Michmelia*) *seminigra*	11	_	[45]
*Meliponula bocandei*	18	_	[85]
*Meliponula ferruginea*	18	_	[85]
*Mourella caerulea*	17	11ª+6A^m^	[56]
*Nannotrigona testaceicornis*	17	18A+16A^m^	[11], [56], [58]
*Oxytrigona tataira*	17		[55], [75]
*Paratrigona subnuda*	17	24A+10A^m^	[56], [58], [75], [78],
*Partamona auripennis*	17	_	Unpublished data
*Partamona testacea*	18	_	[78]
*Plebeia droryana*	17	_	[11], [58], [75], [78], [86]
*Plebeina hildebrandti*	18	_	[77], [78]
***Continuation***			
**Species**	***n***	**Karyotypic formula**	**References**
*Ptilotrigona lurida*	11	6M+3A+2A^m^	[56]
*Scaptotrigona bipunctata*	17	2A+30A^m^+2M	[87]
*Scaptotrigona depilis*	17	26A+8A^m^	[56], [81]
*Scaura latitarsis*	17	1M+2A+14A^m^	[25], [56], [81], [82], [88]
*Scaura longula*	17	3A+14A^m^	[88]
*Schwarziana quadripunctata*	17	18A+16A^m^	[56], [58], [81]
*Tetragona clavipes*	17	6A+28A^m^	[56], [81]
*Tetragonisca angustula*	17	34A^m^	[11], [56], [57], [58], [81]
*Trigona chanchamayoensis*	17	18ª+2A^e^+12A^m^	[22]
*Trigona cilipes*	18	_	[77]
*Trigona fuscipennis*	17	_	[81], [89]
*Trigona hyalinata*	17	4A+2A^e^+30A^m^	[22], [88]
*Trigona hypogea*	17	2A+24A^m^+8A^mc^	[90]
*Trigona pallens*	17	2A^e^+32A^m^	[90]
*Trigona recursa*	17	12A+2A^e^+30A^m^	[56], [81], [90]
*Trigona spinipes*	17	6A+28A^m^	[56], [81], [90]
*Trigona truculenta*	17	4A^e^+24A^m^+6A^mc^	[90]
*Trigona williana*	17	2A^e^+2A+30A^m^	[90]

The same data matrix from phylogenetic analyses was used for molecular dating according to methods previously described by Rasmussen and Cameron [7]. Briefly, the divergence times were estimated using the Bayesian relaxed clock uncorrelated lognormal method implemented in BEAST 2.0 [36] on the CIPRES server [31]. This is the most suitable model for Hymenoptera since it allows evolutionary rates to vary between trees branches [37]. The nucleotide substitution model was GTR+G+I for all partitions and the Yule process was used as *a priori* probability for the trees [38]. We used 300,000,000 generations of MCMC and the convergence was checked in Tracer v.1.7 [34]. A maximum clade credibility tree was created in the program TreeAnnotator v2.4.1 (implemented in BEAST) using 25% burn-in, and was visualized and edited in FigTree v.1.3.1 [35]. Calibration points were based on previous work by Rasmussen and Cameron [7] and Martins *et al*. [39].

### Reconstruction of the ancestral state

In order to evaluate the ancestral chromosome number of Meliponini and further test the fission, fusion, or duplication hypothesis of karyotype evolution in this group of bees, we used three phylogenetic approaches to ancestral reconstruction to estimate the potential ancestral chromosome number.

First, the ancestral chromosome number was reconstructed using Maximum Parsimony (MP) and Maximum Likelihood (MLm) analyses performed with Mesquite software v.3.04 [40]. For these analyses, either the last 1000 trees from the Bayesian MCMC analyses, or the dated phylogeny, were used as the input. In both analyses, the different haploid numbers (*n*) of each species were considered as character states (S2 Table), and the values of the ancestral chromosome number, the most parsimonious state(s) in MP, were represented by percentages (%) in the MLm analysis.

Second, we performed additional analysis with a different methodology to evaluate the consistency of the recovered data. We estimated the ancestral haploid chromosome number of the Meliponini and sister group in three independent analyses using Chromevol 2.0 [41], which on the basis of molecular phylogeny estimates the haploid ancestral chromosome number by using two probabilistic methods, maximum likelihood (ML) and Bayesian inference (BI), with the latter providing a posterior probability. Chromevol 2.0 can evaluate ten chromosome evolution models and different transitions between chromosome numbers. The models evaluate dysploidy (under constant or linear rates), polyploidy (duplication), and demi-polyploidy (demi-duplication), thus testing the possibility of changes in the karyotype that result from changes in ploidy, and also the null model in each case for no duplication. All parameters were adjusted for the data, as described by Glick and Mayrose [41], Cristiano et al. [42] and Cardoso et al. [43]. The model that fits best was analyzed with 10,000 simulations under the AIC.

## Results

### Chromosome number, phylogenetic analyses, and molecular dating

Meliponini species showed variation of haploid number ranging from *n* = 8 to *n* = 18 chromosomes, with *n* = 17 being the predominant chromosome number. The Old World species presented only *n* = 17 and *n* = 18 chromosomes, and in the New World species the number of chromosomes ranged from *n* = 8 to *n* = 18. In Bombini species, on the other hand, the haploid number varied from *n* = 12 to *n* = 20 chromosomes, with *n* = 18 predominating (Table 1).

The concatenated dataset resulted in 3,263 aligned base pairs and the phylogenetic tree obtained from Bayesian inference analysis recovered the phylogeny proposed by Rasmussen and Cameron [7] (S1 Fig). According to this phylogeny, the Old World clade is formed by the Meliponini of the Afrotropical, Australasian, and Indo-Malayan regions, and the New World clade is formed by the species of the Neotropical region. The Neotropical Meliponini initially diverged into two clades, separating *Trigonisca* sensu lato (clade *Trigonisca* s.l.) which includes the genera *Dolichotrigona* (Moure, 1950), *Trigonisca* (Moure, 1950), *Celetrigona* (Moure, 1950), and *Leurotrigona* (Moure, 1950) from the remaining species. Subsequently, there was a second split between *Melipona* sensu lato (*Melipona* s.l.) and the other Meliponini (also see Rasmussen and Cameron [7]).

**Fig 1:**
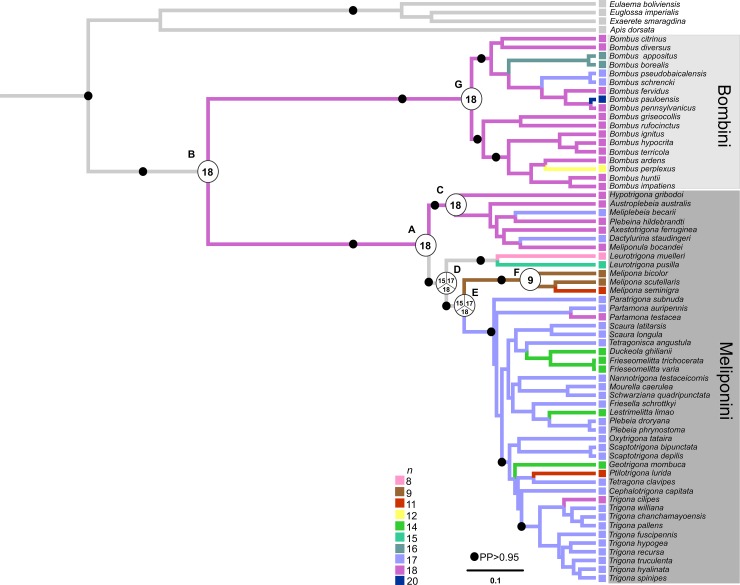
Consensus tree obtained from the Bayesian analysis of concatenated data based on partial sequences of the Arg-K, Opsin, EF1-α, 28S and 16S genes from Meliponini and Bombini species, and ancestral chromosome number inference as implemented in Mesquite by MP analysis. The squares in the terminal branches and the color of the branches represent the different haploid numbers, and the ancestor nodes indicate the ancestral states estimated to be the most parsimonious.

According to molecular dating, the most recent common ancestor between Bombini and Meliponini is dated to about 79.1 (95% HPD = 74–83.3) million years ago (mya) in the upper Cretaceous. Among the Meliponini, the common ancestor dates to about 65.5 (95% HPD = 65–66.6) mya, corresponding to the Paleocene, and, between species of the genus *Melipona*, to about 18.1 (95% HPD = 12–26) mya, corresponding to the Miocene (Fig 1; S2 Fig).

### Reconstruction of the ancestral chromosome number

**Fig 2:**
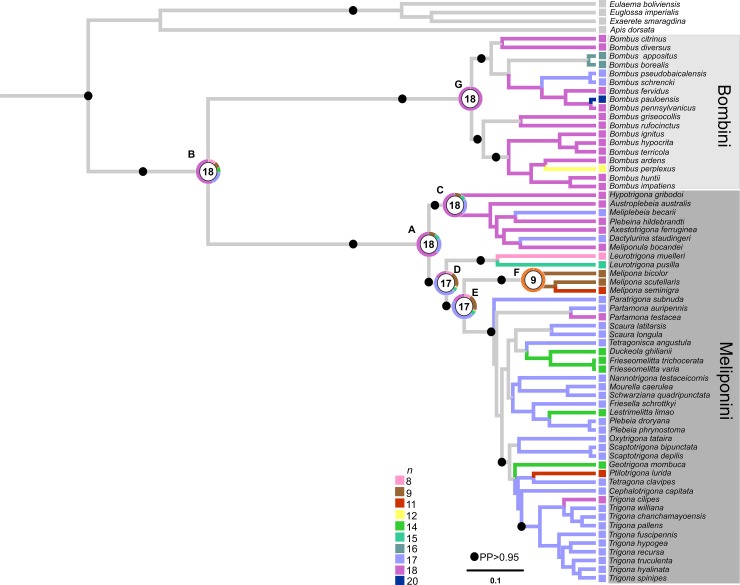
Consensus tree obtained from the Bayesian analysis of concatenated data based on partial sequences of the Arg-K, Opsin, EF1-α, 28S and 16S genes from Meliponini and Bombini species, and ancestral chromosome number inference as implemented in Mesquite by ML analysis. The squares in the terminal branches and the color of the branches represent the different haploid numbers, and the ancestor nodes indicate the most likely ancestral state. Pie charts indicate the probabilities of each ancestral state.

The ancestral reconstruction performed in Mesquite, which considered both the phylogram and the chronogram using both MP and MLm, indicated *n* = 18 as the ancestral chromosome number for the Meliponini tribe (73%, node A), and *n* = 18 (75%, node B) as the ancestral chromosome number for Meliponini and Bombini (Fig 1 and Fig 2). In Meliponini species belonging to the Old World clade, *n* = 18 chromosomes remained in most of the lineages (97%, node C), whereas there was a reduction from *n* = 18 (37%, node D) to *n* = 17 chromosomes (50%, node E) in the New World clade. One exception was *Melipona*, which experienced a reduction to half the number of chromosomes (from *n* = 18 to *n* = 9) (100%, node F). In Bombini, *n* = 18 chromosomes remained the most common number (100%, node G), with a reduction to *n* = 17 and *n* = 16 chromosomes in the subgenera *Subterraneobombus* and *Thoracobombus*, respectively. All values referring to the probabilities of each character found in the ancestor nodes of the Meliponini and Bombini species are indicated in the Appendix (S2 Table).

**Fig 3:**
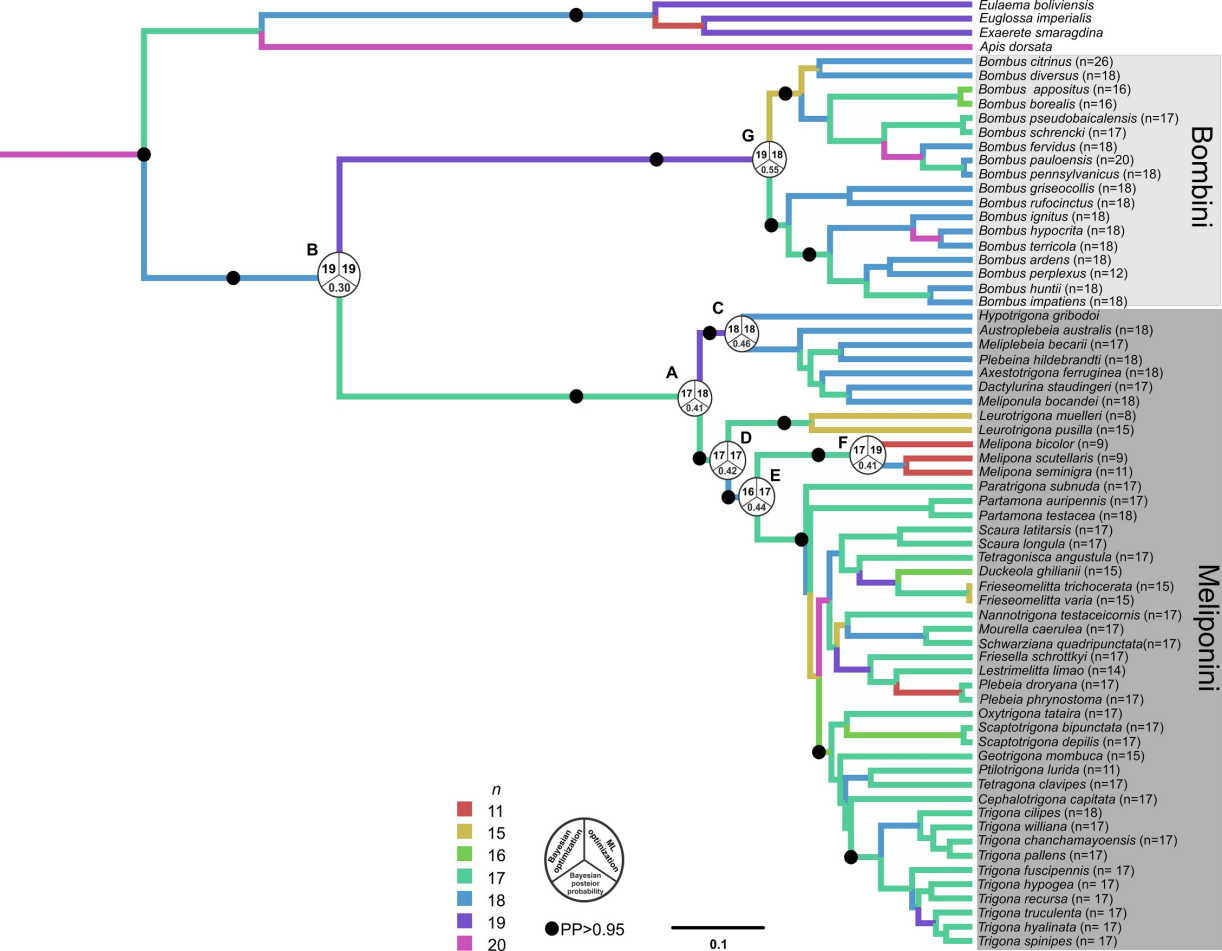
Consensus tree obtained from the Bayesian analysis of concatenated data based on partial sequences of the Arg-K, Opsin, EF1-α, 28S and 16S genes from Meliponini and Bombini species, including ancestral haploid chromosome state reconstruction inferred under Bayesian and Maximum Likelihood optimizations in Chromevol 2.0 software. Pie charts at nodes represent the inferred chromosome number in both Maximum Likelihood optimization and the first data for Bayesian optimization and its Bayesian posterior probabilities.

The reconstruction using ML and BI optimization in Chromevol 2.0, performed using the same trees, also recovered ancestral haploid numbers around 17, 18, and 19 chromosomes (Fig 3), considering the linear rate with no duplication model (AIC = 254, Likelihood = -123). As with ML analysis implemented in Mesquite, ML optimization on Chromevol 2.0 also found *n* = 18 to be the ancestral chromosome number for the Meliponini tribe (node A), but determined *n* = 19 (node B) to be the ancestral chromosome number for Meliponini and Bombini. Meliponini species belonging to the Old World clade were found to have *n* = 18 chromosomes in node C, whereas *n* = 17 chromosomes was determined for in the New World clade in nodes D and E. Yet for the *Melipona* genus, *n* = 11 was recovered instead of *n* = 9 (node F), while *n* = 18 chromosomes was identified for Bombini. Results from Bayesian optimization in Chromevol 2.0 were very similar to those generated by ML optimization, recovering the same ancestral chromosome number in one out of the two estimates with the highest posterior probability (Table 2).

**Table 2:** Haploid ancestral chromosome number recovered by the different methods implemented in Mesquite 3.04 and Chromevol 2.0.

Nodes	Estimated Haploid Ancestral Chromosome Number				
	Maximum Parsimony in Mesquite	Maximum Likelihood in Mesquite (%)	Maximum likelihood optimization in Chromevol 2.0	Bayesian optimization in Chromevol 2.0	
				1^st^ highest P.P. estimate (P.P.)	2^nd^ highest P.P. estimate (P.P.)
A–Meliponini	18	18 (52)	18	17 (0.41)	18 (0.34)
B–Meliponini plus Bombini	18	18 (42)	19	19 (0.30)	20 (0.25)
C–Old World Meliponini	18	18 (99)	18	18 (0.46)	17 (0.32)
D–New World Meliponini	15/ 17/18	18 (31)	17	17 (0.42)	16 (0.39)
E–*Melipona* plus remaining Meliponini	15/ 17/ 18	17 (45)	17	16 (0.44)	17 (0.41)
F–*Melipona*	9	9 (100)	11	11 (0.40)	12 (0.39)
G–Bombini	18	18 (100)	18	19 (0.55)	18 (0.31)

## Discussion

This is the first study reconstructing the ancestral chromosome number in Meliponini based on cytogenetic and molecular data by means of distinct and complementary approaches. Our results indicate that the most likely common ancestor of the Meliponini tribe had *n* = 18 chromosomes and that, in the Neotropical species, this chromosome number decreased to *n* = 17. According to karyotype descriptions, Meliponini can be separated into three groups based on the most frequent number of chromosomes in the species (reviewed in Tavares *et al*. [10]). The first group consists of Meliponini species with *n* = 17 chromosomes. Although different species have the same chromosome number (*n* = 17), the morphological variation observed in the karyotypes (Table 1) indicates that rearrangements such as inversions and translocations were responsible for variations in chromosome structure [16], [25], [41]. A variation in the number of chromosomes was observed in *Trigona* sp., possibly *Trigona braueri* (Friese, 1900) (described as *Trigona fulviventris* Guérin, 1844 in Domingues *et al*.[44]) with 2*n* = 32 chromosomes, unlike the other *Trigona* species with 2*n* = 34. This reduction of the chromosome number is the result of centric fusion of two pseudoacrocentric chromosomes, which generated a larger metacentric chromosome with heterochromatin restricted to the pericentromeric region [44].

The second group is formed by species with *n* = 15 chromosomes, a chromosomal number which would have appeared independently several times during the evolution of Meliponini. The third group is composed of species of the genus *Melipona* that typically have *n* = 9 chromosomes. This low chromosome number is apomorphic for this group, and departures from this basic number are known variations particular to this genus. *Melipona seminigra* Friese, 1903 (*n* = 11) is one exception whose chromosome number could have arisen by fission from an ancestor with *n* = 9 [45]. Yet, *Melipona quinquefasciata* (Lepeletier, 1836) and *Melipona rufiventris* (Lepeletier, 1836) sometimes demonstrate a karyotype with more than 9 chromosomes due to the presence of chromosomes B, which are not part of complement A [46–47]. B chromosomes are expendable elements found together with the chromosome set (complement A) in some specimens belonging to different taxa [48–49]. These chromosomes are characterized by a non-Mendelian inheritance pattern, as they do not undergo recombination due to their lack of homology with complement A chromosomes. Repetitive DNA sequences are generally enriched in B chromosomes, especially those associated with satellite DNA, ribosomal DNA (rDNA) and transposable elements [48–52].

Initial studies in bees revealed that some species have a low chromosome number, between *n* = 8 and *n* = 9 [11], [53–54], and that the pattern of heterochromatin distribution within chromosomes is similar to that observed in ant species of the genus *Myrmecia* (Fabricius, 1804) [22], [55–57],. Using cytogenetic data collected from the *Myrmecia pilosula* complex, Imai *et al*. [18], [19], [20] observed that the ancestor of this group had a lower chromosome number when compared to species that had recently diverged. They also observed that there was an increase in heterochromatin in one of the chromosome arms in the species with the highest diploid number. Thus, considering the cytogenetic information and phylogenetic relationships between these species, they proposed that the ancestral karyotype of this group should have a low chromosome number (*i*.*e*. *n* = 3) and that centric fissions would be the main rearrangement responsible for the increase in chromosome number [18–20]. Such cytogenetic patterns led the researchers to suggest that the same mechanism would be involved in chromosome evolution in bees, and that the ancestral species would have a chromosome number smaller than that found in species that diverged more recently [11], [22–25], [54–55], [58–59]. However, our analysis indicates that the ancestral karyotype of Meliponini had a high chromosomal number (*n* = 18), which was maintained in many species, and that, possibly as a result of fusion events, this number decreased from *n* = 18 to *n* = 17 in the Neotropical Meliponini, contrary to the expected pattern indicated by the MIT for chromosome evolution in bees. According to the theory, modifications in the karyotypes that occur through centric fission in different species occur in order to minimize the deleterious effects of chromosomal interactions [19–21].

In addition to a decrease from the ancestral chromosome number in the Meliponini, some structural characteristics of the chromosomes of from *Melipona* species also suggest that this group does not follow the evolutionary model proposed by MIT. Species of *Melipona* have unique characteristics that distinguish them from other Meliponini species, such as a caste differentiation system that is based on genetic characteristics shaped by the environment rather than the amount of food received [60], [61], and phylogenetically, the genus is monophyletic in relation to the other Neotropical Meliponini [7], [62], [63]. Furthermore, cytogenetically the species present a haploid number of nine chromosomes and the genus is subdivided into two groups characterized by the spatial distribution of heterochromatin along the chromosome arms. In Group I, heterochromatin is observed in the pericentromeric region, whereas in Group II, it is dispersed evenly along most chromosomes [54–56].

Phylogenetic reconstructions and the time of divergence suggest that the *Melipona* species diverged more recently (± 20 Ma) than those Meliponini with a higher number of chromosomes (± 54 Ma) [7]. Thus, the unique characteristics of the genus in relation to its divergence time suggest that *Melipona* followed a "different" pattern from the other Meliponini, and underwent different evolutionary processes that were different from the remaining species of this tribe. Thus, given there has been about 20 million years of divergence from the time of the common *Melipona* ancestor, we believe that repetitive centric fusions were responsible for the decreasing the chromosome number. Further changes in karyotypic structure may be the outcome of inversions, translocations, and the repositioning of transposable elements.

Centric fusion is considered one of the major chromosomal rearrangements in animal karyotype evolution [64]. Rearrangements of this type were used to explain the karyotype evolution in wasps of the Epiponini tribe [65], parasitic wasps (*Minotetrastichus frontalis* (Nees, 1834) and *Chrysocharis laomedon* (Walker, 1839) [66], and ants (*Mycetophylax morschi* (Emery, 1888)) [43]. In other taxonomic groups, fusions have also been suggested as the main mechanism responsible for changes in chromosome numbers, as in locusts of the Ephippigerini tribe [67], and in several species of mammals (*Elaphodus cephalophus* (Milne-Edwards, 1873), *Muntiacus reevesi* (Ogilby, 1839) and *Muntiacus muntjak vaginalis* (Boddaert, 1785)) [68–71].

On the other hand, in different taxa such as ants, fish, mammals, and frogs, fissions are also important events in chromosome rearrangement throughout evolutionary time [21], [72–74]. In Meliponini, an example of chromosome fission was observed in *Melipona seminigra* (Friese, 1903), which has *n* = 11 chromosomes [12], [45]. According to our findings, this chromosome number observed today likely originated by fission events from an ancestor with *n* = 9. Similar events may have shaped chromosome number evolution in *Trigona cilipes* (Fabricius, 1804), so that fission in an ancestor with *n* = 17 led to the karyotype with *n* = 18 chromosomes. However, chromosome fission requires the formation of new centromeres and telomeres for the new chromosomes [75], and therefore may not be the most common mechanism in karyotype evolution in different groups.

The results of this study, with cytogenetic evidence and ancestral states, also suggest that the ancestor between Meliponini and Bombini had *n* = 18 chromosomes. Cytogenetic descriptions found for the other corbiculate tribes show a range in chromosome number between *n* = 8 and *n* = 21. For example, Apini (*n* = 17) ([1]), Euglossini (*n* = 20–21), Bombini (*n* = 18–20) [11], [2], [8], and Meliponini (*n* = 8–18, with the most common being *n* = 17) [10], [76]. Owen *et al*. [2] considered the ancestral number to be *n* = 18 for *Bombus*, and that variations of *n* = 16 (*Bombus* (*Subterraneo*) *appositus* (Cresson, 1878) and *Bombus* (*Subterraneo*) *borealis* (Kirby, 1837), *n* = 17 (*Bombus* (*Thoracobombus*) *pseudobaicalensis* (Vogt, 1911) and *Bombus* (*Thoracobombus*) *schrenck* (Morawitz, 1881) and *n* = 20 (*Bombus* (*Thoracobombus*) *pauloensis* (Friese, 1913)) would be the result of chromosomal fusions and fissions. Although the Meliponini and Bombini species have similar ancestral chromosome numbers, the Meliponini have diploid numbers, chromosome morphologies, and heterochromatin distribution patterns conserved among species, differently from Bombini, which show variations in these cytogenetic patterns. Our results suggest that the ancestor of the Bombini tribe had a high chromosomal number (*n* = 18), and that this chromosome number was maintained throughout evolution in several species, which contradicts what was expected from MIT [11].

Based on the cytogenetic information, as well as on insights into chromosome evolution using a phylogenetic approach in Meliponini, we propose here that the ancestral chromosome number between the Meliponini and Bombini tribes is *n* = 18 chromosomes. This chromosome number remained in the common ancestor of Meliponini, and by Robertsonian chromosomal fusion, decreased from *n* = 18 to *n* = 17 in the Neotropical Meliponini. Yet, the low number of chromosomes found in *Melipona* is an apomorphy of that clade likely due chromosomal fusions. We also conclude that chromosome fissions, as predicted by MIT, are not the main mechanism in karyotype evolution of Meliponini and Bombini. It was more likely that the ancestral chromosome number (i.e. *n* = 18) was maintained across bee lineages, and that it is equally possible for the variation in haploid chromosome number to have arisen by chromosomal fusion and fission.

## Supporting information

S1 Fig Consensus tree of Bayesian analysis, based on partial sequences of the Arg-K, Opsin, EF1-α, 28S and 16S concatenated genes of the Meliponini and Bombini. The numbers after the nodes represent the later probabilities, blue branches represent the tribe Meliponini, while green branches indicate Bombini. The outgroups were represented by *Exaerete smaragdina*, *Eulaema boliviensis* and *Euglossa imperialis*.
(TIFF)

S2 Fig Consensus tree of Bayesian analysis based on partial sequences of the Arg-K, Opsin, EF1-α, 28S and 16S concatenated genes of the Meliponini and Bombini species including the times of divergence estimated in the Beast program. The bars indicate 95% confidence. Outgroups were represented by *Exaerete smaragdina*, *Eulaema boliviensis* and *Euglossa imperialis*.
(TIF)

S1 Table Species of bees and the external group analyzed, collection site, gene access number in GenBank (http://www.ncbi.nlm.nih.gov) and references. (DOCX)

S2 Table Probabilities (in percentages) of the haploid numbers in the reconstruction of the ancestral state between the clades. (DOCX)

## References

[1] Hoshiba H, Kusanagi A. Karyological study of honeybee. J Apic Res. 1978;17: 105–109.

[2] Owen RE, Richardfs KW, Wilkes A. Chromosome numbers and karyotypic variation in Bumble bees (Hymenoptera: Apidae; Bombini). J Kans Entomol Soc. 1995;68: 290–302.

[3] Hines HM, Cameron SA, Williams PH. Molecular phylogeny of the bumble bee subgenus *Pyrobombus* (Hymenoptera: Apidae: *Bombus*) with insights into gene utility for lower-level analysis. Invertebr Syst. 2006;20: 289–303.

[4] Cameron SA, Hines HM, Williams PH. A comprehensive phylogeny of the bumble bees (Bombus). Biol J Linn Soc. 2007;91: 161–188.

[5] Michener CD. The Bees of the World The John Hopkins University Press, London 2007.

[6] Camargo JMF, Pedro SEM. Meliponini Lepeletier, 1836. In Moure J.S., Urban D., Melo G.A.R. (Orgs). Catalogue of Bees (Hymenoptera, Apoidea) in the Neotropical Region—online version. 2013 Available at http://www.moure.cria.org.br/catalogue. Last Accessed 6/06/2018).

[7] Rasmussen C, Cameron SA. Global stingless bee phylogeny supports ancient divergence, vicariance, and long distance dispersal. Biol J Linn Soc Lond. 2010;99: 206–232.

[8] Fernandes A, Werneck HA, Pompolo SG, Lopes DM. Evidence of separate karyotype evolutionary pathway in *Euglossa* orchid bees by cytogenetic analyses. An Acad Bras Ciênc. 2013;85: 937–944. 10.1590/S0001-37652013005000050 23969851

[9] Françoso E, Oliveira FF, Arias MC. An interative approach identifies a new pecies of bumblebee (Hymenoptera: Apidae: Bombini) from northeastern Brazil. Apidologie.2016;47: 171–185.

[10] Tavares MG, Lopes DM, Campos LAO. An overview of cytogenetics of the tribe Meliponine (Hymenoptera: Apidae). Genetica. 2017;145: 1–18. 10.1007/s10709-016-9939-5 28315980

[11] Hoshiba H, Imai HT. Chromosome evolution of bees and wasps (Hymenoptera, Apocrita) on the basis of C-banding pattern analyses. Jpn J Entomol. 1993;61: 465–492.

[12] Cunha MS, Travenzoli NM, Ferreira RP, Cassinela EK, Silva H, Salomão TMF, et al Comparative cytogenetics in three *Melipona* species (Hymenoptera: Apidae) with two divergent heterochromatic patterns. Genet Mol Biol. 2018;4: 806–813. 10.1590/1678-4685-GMB-2017-0330 PMC6415597 30508005

[13] Cristiano MP, Simões TG, Lopes DM, das Graças Pompolo S. Cytogenetics of *Melitoma segmentaria* (Fabricius, 1804) (Hymenoptera, Apidae) reveals differences in the characteristics of heterochromatin in bees. Comp Cytogenet. 2014;8: 223 10.3897/CompCytogen.v8i3.7510 25349673 PMC4205491

[14] Brito RM, Pompolo SG, Magalhães MFM, Barros EG, Sakamoto-Hojo ET. Cytogenetic characterization of two *Partamona* species (Hymenoptera, Apidae, *Meliponini*) by fluorochrome staining and localization of 18 S rDNA clusters by FISH. Cytologia. 2005;70: 73–380.

[15] Piccoli MCA, Bardella VB, Cabral-de-Mello DC. Repetitive DNAs in Melipona scutellaris (Hymenoptera: Apidae: Meliponidae): chromosomal distribution and test of multiple heterochromatin amplification in the genus. Apidologie. 2018;1: 8.

[16] Kerr WE, Silveira ZV. Karyotypic evolution of bees and corresponding taxonomic implications. Evol Int J Org Evol. 1972;26: 197–202. 10.1111/j.1558-5646.1972.tb00187.x 28555733

[17] Imai HT. On the origin of telocentric chromosomes in Mammals. J Theor Biol. 1978;71: 619–637. 10.1016/0022-5193(78)90328-4 661326

[18] Imai HT, Maruyama T, Gojobori T, Inoue Y, Crozier RH. Theoretical bases for karyotype evolution. The minimum-interaction hypothesis. Am Nat. 1986;128: 900–920.

[19] Imai HT, Taylor RW, Crosland MW, Crozier RH. Modes of spontaneous chromosomal mutation and karyotype evolution in ants with reference to the minimum interaction hypothesis. J Gen. 1988;63: 159–185. 10.1266/jjg.63.159 3273765

[20] Imai HT, Taylor RW, Crozier RH. Experimental bases for the minimum interaction theory. I. Chromosome evolution in ants of the *Myrmecia pilosula* species complex (Hymenoptera: Formicidae: Myrmeciinae). J Gen. 1994;69: 137–182.

[21] Imai HT, Satta Y, Takahata N. Integrative study on chromosome evolution of mammals, ants and wasps based on the minimum interaction theory. J Theor Biol. 2001;210: 475–497. 10.1006/jtbi.2001.2327 11403567

[22] Costa KF, Brito RM, Miyazawa CS. Karyotypic description of four species of *Trigona* (Jurine, 1807) (Hymenoptera, Apidae, Meliponini) from the State of Mato Grosso, Brazil. Genet Mol Biol. 2004;27: 187–190.

[23] Krinski D, Fernandes A, Rocha MP, Pompolo SDG. Karyotypic description of the stingless bee *Oxytrigona* cf. *flaveola* (Hymenoptera, Apidae, Meliponina) of a colony from Tangará da Serra, Mato Grosso State, Brazil. Genet Mol Biol. 2010;33: 494–498. 10.1590/S1415-47572010000300020 21637423 PMC3036110

[24] Godoy DC, Ferreira RP, Lopes DM. Chromosomal variation and cytogenetics of *Plebeia lucii* and *P. phrynostoma* (Hymenoptera: Apidae). Fla Entomol. 2013;96: 1559–1566.

[25] Rocha MP, Pompolo SG, Campos LAO. Citogenética da tribo Meliponini (Hymenoptera, Apidae) In: Melo GAR, Santos IA (eds) Apoidea Neotropica. 2003b Homenagem aos 90 anos de Jesus Santiago Moure. UNESC, Santa Catarina.

[26] Katoh K, Standley DM. MAFFT: iterative refinement and additional methods. Methods Mol Biol. 2014;1079: 131–146. 10.1007/978-1-62703-646-7_8 24170399

[27] Tamura K, Stecher G, Peterson D, Filipski A, Kumar S. MEGA6: molecular evolutionary genetics analysis version 6.0. Mol Biol Evol. 2013;30: 2725–2729. 10.1093/molbev/mst197 24132122 PMC3840312

[28] Tomita M, Shimizu N, Brutlag DL. Introns and Reading frames: correlation between splicing sites and their codon positions. Genome Biol Evol. 1996;13: 1219–1223. 10.1093/oxfordjournals.molbev.a025687 8896374

[29] Kawakita A, Sota T, Ascher JS, Ito M, Tanaka H, Kato M. Evolution and phylogenetic utility of alignment gaps within intron sequences of three nuclear genes in bumble bees (*Bombus*). Mol Biol Evol. 2003;20: 87–92. 10.1093/molbev/msg007 12519910

[30] Vaidya G, Lohman DJ, Meier R. Sequence Matrix: concatenation software for the fast assembly of multigene datasets with character set and codon information. Cladistics. 2011;27: 171–180. 10.1111/j.1096-0031.2010.00329.x 34875773

[31] Miller MA, Pfeiffer W, Schwartz T. "Creating the CIPRES Science Gateway for inference of large phylogenetic trees" in Proceedings of the Gateway Computing Environments Workshop (GCE). 2011. New Orleans, LA.

[32] Ronquist F, Klopfstein S, Vilhelmsen L, Schulmeister S, Murray DL, Rasnitsyn AP. A total-evidence approach to dating with fossils, applied to the early radiation of the Hymenoptera. Syst Biol. 2012;61: 973–99. 10.1093/sysbio/sys058 22723471 PMC3478566

[33] Ronquist F, Huelsenbeck JP. MrBayes 3: Bayesian phylogenetic inference under mixed models. Bioinformatic. 2003;19: 1572–1574. 10.1093/bioinformatics/btg180 12912839

[34] Rambaut A, Drummond A. Tracer, Version 1.7. http://tree.bio.ed.ac.uk/software/tracer/. 2009. Last accessed 22/08/2019.

[35] Rambaut A. FigTree, ver. 1.3.1. [Online]. Available: http://tree.bio.ed.ac.uk/software/ figtree/. 2009. Last accessed 02/05/2018.

[36] Bouckaert R, Heled J, Kühnert D, Vaughan T, Wu C-H, Xie D, et al BEAST 2: A Software Platform for Bayesian Evolutionary Analysis. PLoS Comput Biol. 2014;10: e1003537 10.1371/journal.pcbi.1003537 24722319 PMC3985171

[37] Drummond AJ, Ho SYW, Phillips MJ, Rambaut A. Relaxed phylogenetics and dating with confidence. PloS Biol. 2006;4: 699–710. 10.1371/journal.pbio.0040088 PMC1395354 16683862

[38] Drummond AJ, Rambaut A. BEAST: Bayesian evolutionary analysis by sampling trees. BMC Evol Biol. 2007;7: 214 10.1186/1471-2148-7-214 17996036 PMC2247476

[39] Martins AC, Melo GAR, Renner SS. The corbiculate bees arose from New World oil-collecting bees: Implications for the origin of pollen baskets. Mol Phylogenet Evol. 2014;80: 88–94. 10.1016/j.ympev.2014.07.003 25034728

[40] Madison WP, Madison DR. Mesquite: A modular system for evolutionary analysis, version 2.75. http://mesquiteproject.org. 2011. Last accessed 02/06/2018.

[41] Glick L, Mayrose I. ChromEvol: Assessing the pattern of chromosome number evolution and the inference of polyploidy along a phylogeny. Mol Biol Evol. 2014;31: 1914–1922. 10.1093/molbev/msu122 24710517

[42] Cristiano MP, Cardoso DC, Fernandes-Salomao TM. Cytogenetic and molecular analyses reveal a divergence between *Acromyrmex striatus* (Roger, 1863) and other congeneric species: taxonomic implications. PloS One. 2013;8: 9. 10.1371/journal.pone.0059784 PMC3603875 23527267

[43] Cardoso DC, Pompolo SG, Cristiano MP, Tavares MG. The role of fusion in ant chromosome evolution: insights from cytogenetic analysis using a molecular phylogenetic approach in the genus *Mycetophylax*. PLoS One. 2014;9: e87473 10.1371/journal.pone.0087473 24489918 PMC3904993

[44] Domingues AMT, Waldschmidt AM, Andrade SE, Andrade-Souza V, Alves RMDO, Silva-Junior JCD, et al Karyotype characterization of *Trigona fulviventris* Guérin, 1835 (Hymenoptera, Meliponini) by C banding and fluorochrome staining: Report of a new chromosome number in the genus. Genet Mol Biol. 2005a;28: 390–393.

[45] Francini IB, Gross MC, Nunes-Silva CG, Carvalho-Zilse GA. Cytogenetic analysis of the Amazon stingless bee *Melipona seminigra merrillae* reveals different chromosome number for the genus. Sci Agric. 2011;68: 592–593.

[46] Lopes DM, Pompolo SDG, Campos LADO, Tavares MG. Cytogenetic characterization of *Melipona rufiventris* Lepeletier 1836 and *Melipona mondury* Smith 1863 (Hymenoptera, Apidae) by C banding and fluorochromes staining. Genet Mol Biol. 2008;31: 49–52.

[47] Silva AA, Rocha MP. Karyotypic description of the stingless bee *Melipona quinquefasciata* Lepeletier, 1836 (Hymenoptera, Meliponini) with emphasis on the presence of B chromosomes. Comp Cytogenet. 2018;12: 471 10.3897/CompCytogen.v12i4.29165 30479700 PMC6240122

[48] Camacho JPM. B Chromosomes. The Evolution of the Genome. 2005; pp.223–286

[49] Houben A, Banaei-Moghaddam AM, Klemme S, Timmis JN. Evolution and biology of supernumerary B chromosomes. Cell Mol Life Sci. 2014; 71: 467–478. 10.1007/s00018-013-1437-7 23912901 PMC11113615

[50] Camacho JPM, Sharbel TF, Beukeboom LW. B-chromosome evolution. Philosophical Transactions of the Royal Society of London. Series B: Biological Sciences. 2000; 355: 163–178. 10.1098/rstb.2000.0556 10724453 PMC1692730

[51] Anjos A, Rocha GC, Paladini A, Mariguela TC, Cabral-de-Mello DC. Karyotypes and repetitive DNA evolution in six species of the genus Mahanarva (Auchenorrhyncha: Cercopidae). Cytogenet Genome Res. 2016; 149: 321–327. 10.1159/000450730 27811473

[52] McAllister BF, Werren JH. Hybrid origin of a B chromosome (PSR) in the parasitic wasp Nasonia vitripennis. Chromosoma. 1996;106: 243–253. 10.1007/s004120050245 9254726

[53] Pompolo SG, Campos LAO. Karyotypes of two species of stingless bees, *Leurotrigona muelleri* and *Leurotrigona pusilla* (Hymenoptera, Meliponinae). Rev Bras Genet. 1995;18: 181–184.

[54] Rocha MP, Pompolo SG. Karyotypes and heterochromatin variation (C-bands) in *Melipona* species (Hymenoptera, Apidae, Meliponinae). Genet Mol Biol. 1998;21: 41–45.

[55] Rocha MP, Pompolo SG, Dergam JA, Fernandes A, Campos LAO. DNA characterization and karyotypic evolution in the bee genus *Melipona* (Hymenoptera, Meliponini). Hereditas. 2002;136: 19–27. 10.1034/j.1601-5223.2002.1360104.x 12184485

[56] Rocha MP, Cruz MP, Pompolo SG, Fernandes A, Silva JCJR, Waldschmidt AM. Longitudinal differentiation in *Melipona mandacaia* (Hymenoptera, Meliponini) chromosomes. Hereditas. 2003a;138: 133–137. 12921165 10.1034/j.1601-5223.2003.01699.x

[57] Barth A, Fernandes A, Pompolo SDG, Costa MA. Occurrence of B chromosomes in *Tetragonisca* Latreille, 1811 (Hymenoptera, Apidae, Meliponini): a new contribution to the cytotaxonomy of the genus. Genet Mol Biol. 2011;34: 77–79. 10.1590/S1415-47572010005000100 21637547 PMC3085378

[58] Pompolo SG. Estudos citogenéticos em Meliponinae. Anais do Encontro Brasileiro sobre Biologia de Abelhas e outros Insetos Sociais. Naturalia. 1992 Ed. Especial, pp 62–66.

[59] Costa MA, Pompolo SG, Campos LAO. Supernumerary chromosomes in *Partamona cupira* (Hymenoptera, Apidae, Meliponinae). Rev Bras Genet. 1992;15: 801–806.

[60] Kerr WE. Estudos sobre o gênero Melipona. Na Esc Super Agric Luiz de Queiroz. 1948;5: 182–276.

[61] Kerr WE, Nielsen RA. Evidence that genetically determined *Melipona* queens can become workers. Genetics. 1966;54: 859–865. 5970624 10.1093/genetics/54.3.859 PMC1211207

[62] Rasmussen C, Cameron SA. A molecular phylogeny of the old word stingless bees (Hymenoptera: Apidae: Meliponini) and the non-monophyly of the large genus *Trigona*. Syst Entomol. 2007;32: 26–39.

[63] Ramírez SR, Nieh JC, Quental TB, Roubik DW, Imperatriz-Fonseca VLI, Pierce NE. A molecular phylogeny of the stingless bee genus *Melipona* (Hymenoptera: Apidae). Mol Phylogenet Evol. 2010;56: 519–525. 10.1016/j.ympev.2010.04.026 20433931

[64] White MJD. Animal cytology and evolution 1973 3rd ed Cambrige University Press.

[65] Menezes RST, Carvalho JPSO, Silva TS, Somovilla A, Costa MA. Evolutionary trends in the chromosome numbers of swarm-founding social wasps. Insec Soc. 2014;61: 385–393.

[66] Gokhman VE. Karyotype Evolution in Parasitic Wasps (Hymenoptera). Zool Zhurnal. 2004;83: 961–970.

[67] Warchałowska-Śliwa E, Grzywacz B, Heller KG, Chobanov DP. Comparative analysis of chromosomes in the Palaearctic bush-crickets of tribe Pholidopterini (Orthoptera, Tettigoniinae). Comp Cytogenet. 2017;11: 309 10.3897/CompCytogen.v11i2.12070 28919967 PMC5596980

[68] Wang W, Lan H. Rapid and parallel chromosomal number reductions in muntjac deer inferred from mitochondrial DNA phylogeny. Mol Biol Evol. 2000;17: 1326–1333. 10.1093/oxfordjournals.molbev.a026416 10958849

[69] Hartmann N, Scherthan H. Characterization of ancestral chromosome fusion points in the *Indian muntjac* deer. Chromosoma. 2004;112: 213–220. 10.1007/s00412-003-0262-4 14648169

[70] Chi JX, Huang L, Nie W, Wang J, Su B, Yang F. Defining the orientation of the tandem fusions that occurred during the evolution of *Indian muntjac* chromosomes by BAC mapping. Chromosoma. 2005;114: 167–172. 10.1007/s00412-005-0004-x 16010580

[71] Tsipouri V, Schueler MG, Hu S, Dutra A, Pak E, Riethman H, et al Comparative sequence analyses reveal sites of ancestral chromosomal fusions in the *Indian muntjac* genome. Genome Biol. 2008;9: R155 10.1186/gb-2008-9-10-r155 18957082 PMC2760882

[72] Imai HT, Crozier RH, Taylor RW. Karyotype evolution in Australian ants. Chromosoma. 1977;59: 341–393.

[73] Busin CS, Vinciprova G, Recco-Pimentel SM. Chromosomal rearrangements as the source of variation in the number of chromosomes in *Pseudis* (Amphibia, Anura). Genetica. 2000;110: 131–141. 10.1023/a:1017957716678 11678503

[74] Primo CC, Glugoski L, Almeida MC, Zawadzki CH, Moreira-Filho O, Vicari MR, et al Mechanisms of chromosomal diversification in species of *Rineloricaria* (Actinopterygii: Siluriformes: Loricariidae). Zebrafish. 2017;14: 161–168. 10.1089/zeb.2016.1386 28027029

[75] Robinson TJ, Ruiz-Herrera A, Froenicke L. Dissecting the mammalian genome–new insight into chromosomal evolution. Trends Genet. 2006;22: 297–301. 10.1016/j.tig.2006.04.002 16678302

[76] Andrade-Souza V, Duarte OMP, Martins CCC, Santos MGC, Costa MA. Comparative molecular cytogenetics of *Melipona* Illiger species (Hymenoptera: Apidae). Sociobiology. 2018;65: 696–705.

[77] Kerr WE. Some aspects of the Evolution of social bees. Evol Biol. 1969;3: 119–175.

[78] Kerr WE. Numbers of chromosomes in some species of bees. J Kansas Entomol Soc. 1972;45: 11–122.

[79] Mampumbu AR. Análise citogenética da heterocromatina e da NOR em populações de abelhas sem ferrão *Friesella schrottkyi* (Friese, 1900) Hymenoptera: Apidae: Meliponini). Dissertation. Universidade Estadual de Campinas UNICAMP, Campinas, Brasil. 2002. Available from: http://repositorio.unicamp.br/jspui/handle/REPOSIP/317994.

[80] Nascimento S. Caracterização citogenética da espécie *Frieseomelitta trichocerata* Moure, 1988 (Hymenoptera; Apidae; Meliponina) coletada em Tangará da Serra-MT Monography, Universidade do Estado de Mato Grosso 2005.

[81] Tarelho ZVS. Contribuição ao estudo citogenético dos Apoidea. Dissertation. Universidade de São Paulo, Ribeirão Preto. 1973.

[82] Silveira ZV. Número de cromossomos em meliponídeos brasileiros. Ciênc Cult. 1971;23: 105–106.

[83] Lopes DM, Fernandes A, Praça-Fontes MM, Werneck HA, Resende HC, Campos LAO. Cytogenetics of three *Melipona* species (Hymenoptera, Apidae, Meliponini). Sociobiology. 2011;58: 185–194.

[84] Hoshiba H. Karyological analysis of a stingless bee, Melipona favosa (Apidae, Hymenoptera). Cytologia, 1988;53: 153–156.

[85] Kerr WE, Araújo VP. Contribuição ao estudo citológico dos Apoidea. I. Espermatogênese em três espécies africanas. Garcia de Orta. 1957;3: 431–433.

[86] Caixeiro AP. Caracterização citogenética da heterocromatina constitutiva e sua implicação na evolução do cariótipo de espécies do gênero *Plebeia* (Hymenoptera: Apinae: Meliponini). Dissertation. Universidade Federal de Viçosa. 1999. Available from: http://www.scielo.br/scielo.php?script=sci_nlinks&ref=000074&pid=S1415-4757200300010000900010&lng=pt.

[87] Godoy DC, Lopes DM, Ferreira RP. Caracterização cariotípica de duas espécies de Meliponini da região Amazônica. In: Anais do Simpósio de Integração Acadêmica de 2014 da Universidade Federal de Viçosa. Viçosa, Minas Gerais. 2014.

[88] Domingues AMT. Estudos citogenéticos comparativos entre espécies de *Scaura* (Hymenoptera, Apidae, Meliponini). Dissertation. Universidade Estadual de Santa Cruz. 2005b. Available from: http://nbcgib.uesc.br/genetica/admin/images/files/OLIVIA%20DUARTE.pdf.

[89] Waldschmidt AM, Duarte OMP, Martins CCC, Santana SEA, Miranda EA, Alves RNO, et al Análises citogenéticas em espécies de abelhas da subtribo Meliponina (Hymenoptera: Meliponina) da região sudoeste da Bahia. In: Anais do 51° Congresso Brasileiro de Genética. Águas de Lindoia, São Paulo, p 253 2005.

[90] Ferreira RP. Análise citogenética de abelhas do gênero *Trigona Jurine*, 1807 (Hymenoptera, Meliponini). Thesis. Universidade Federal de Viçosa. 2015. Available from: http://www.locus.ufv.br/handle/123456789/6495.

