# Supplementary figures and images for: The evolution of haploid chromosome numbers in Meliponini
Source: PLoS One. 2019 Oct 24;14(10):e0224463. doi: 10.1371/journal.pone.0224463 (PMC6812824; doi:10.1371/journal.pone.0224463)

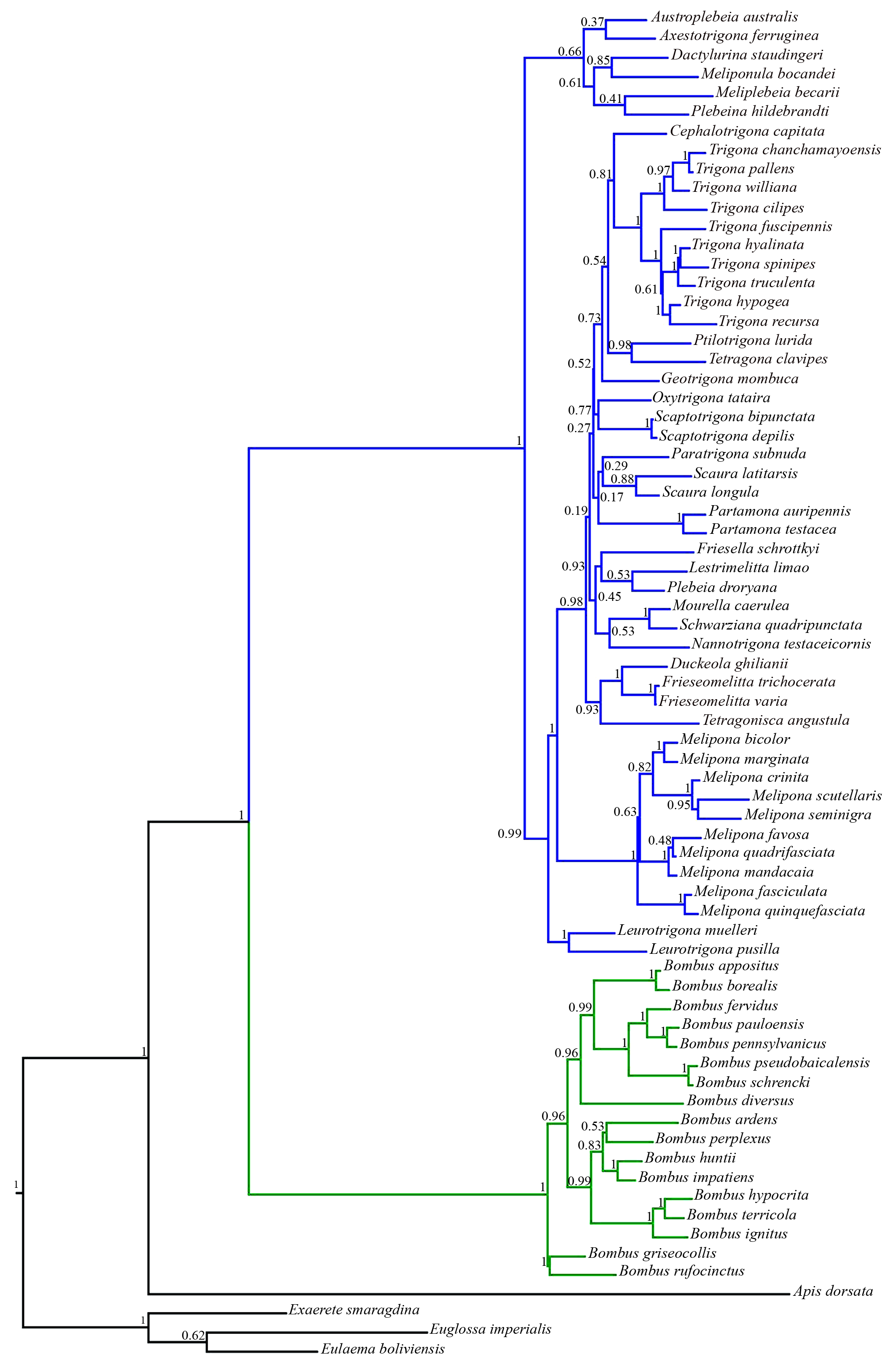

Supplement: S1 Fig — The numbers after the nodes represent the later probabilities, blue branches represent the tribe Meliponini, while green branches indicate Bombini. The outgroups were represented by Exaerete smaragdina, Eulaema boliviensis and Euglossa imperialis. (TIFF) [file pone.0224463.s001.tiff]

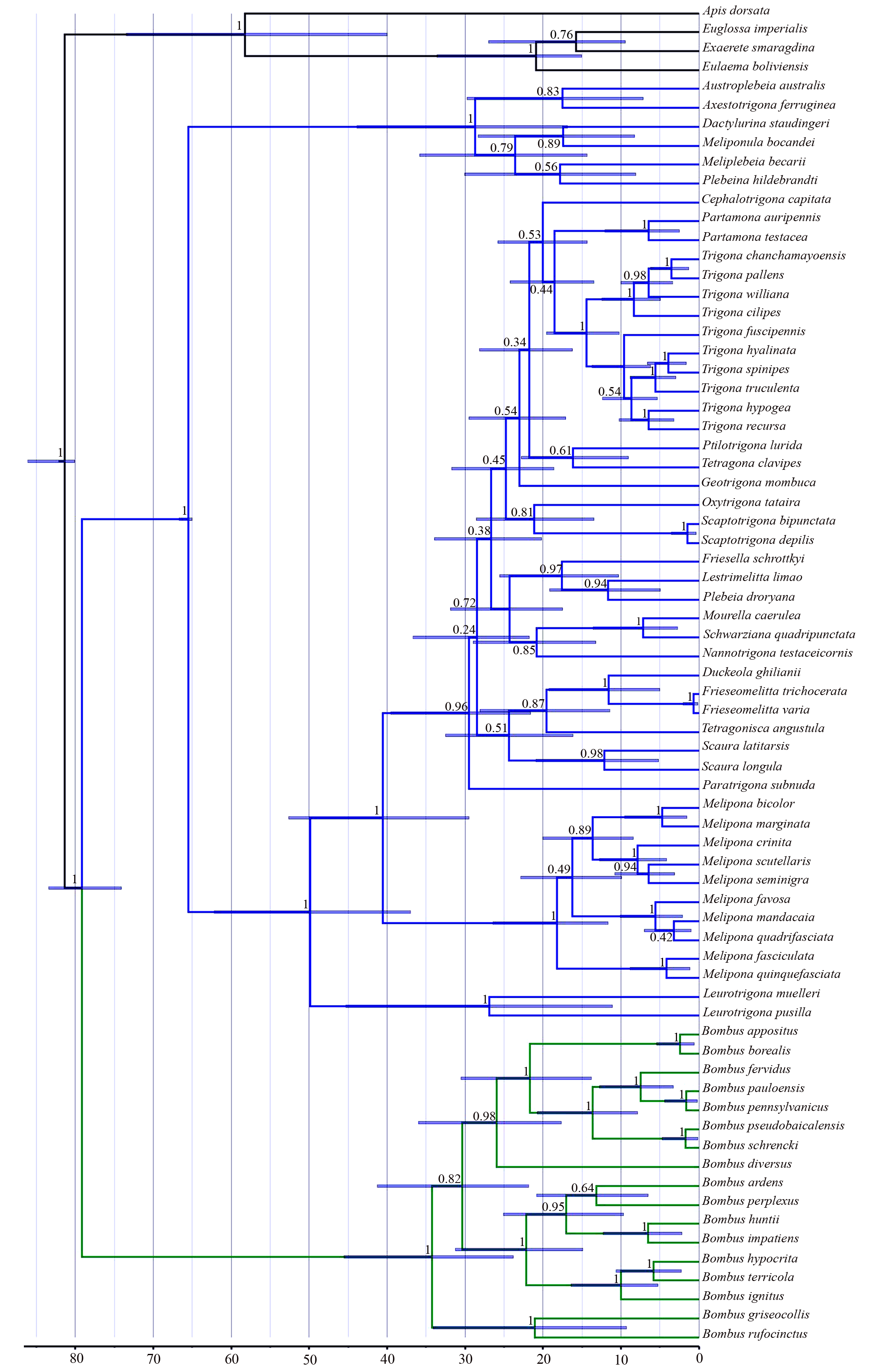

Supplement: S2 Fig — The bars indicate 95% confidence. Outgroups were represented by Exaerete smaragdina, Eulaema boliviensis and Euglossa imperialis. (TIF) [file pone.0224463.s002.tif]
