# Supplementary material for: The evolution of haploid chromosome numbers in Meliponini
Source: PLoS One. 2019 Oct 24;14(10):e0224463. doi: 10.1371/journal.pone.0224463 (PMC6812824; doi:10.1371/journal.pone.0224463)
Supplement: S1 Table — (DOCX) [file pone.0224463.s003.docx]

**S1 Table. Species of bees and the external group analyzed, collection site, gene access number in GenBank (http: //www.ncbi.nlm.nih.gov) and references.**

| **Species** | **Collection site** | **16S** | **ArgK** | **EF1-α** | **Opsin** | **28S** | **References** |
| --- | --- | --- | --- | --- | --- | --- | --- |
| **Ingroup** |  |  |  |  |  |  |  |
| *Austroplebia australis* | Duaringa, Australia | AF343112 | - | - | - | - | [91] |
| *Axestotrigona ferruginea* | Cameroon, Ngaoundere | DQ790450 | DQ813032 | DQ813109 | DQ813187 | FJ042175 | [7] |
| *Bombus* (*Bombus*) *hypocrita* | Kyushu, Japan | AY737347 | AF492889 | JQ820913 | DQ7888209 | - | [3] |
| *Bombus* (*Bombus*) *ignitus* | Beijing, China | AY737348 | AF492898 | DQ788207 | AF493032 | - | [3] |
| *Bombus* (*Bombus*) *terricola* | Ontário, Canada | AY737387 | AF492885 | AY739606 | AF493019 | - | [3] |
| *Bombus* (*Cullumanobombus*) *griseocollis* | Illinois, USA | AY737341 | AF492905 | DQ788196 | AF493039 | - | [3] |
| *Bombus* (*Cullumanobombus*) *rufocinctus* | Alberta, Canada | AY737377 | AF492900 | DQ788268 | AF493034 | - | [3] |
| *Bombus* (*Megabombus*) *diversus* | Kyushu, Japan | DQ788000 | AF492894 | AF492961 | AF493028 | HM750236 | [4], [92] |
| *Bombus* (*Pyrobombus*) *ardens* | Dae-Dong, South Korea | DQ787966 | AF492897 | DQ788149 | AF493031 | HM750237 | [3], [92] |
| *Bombus* (*Pyrobombus*) *huntii* | Washington, USA | AY737344 | AF492911 | DQ788203 | AF493045 | - | [3] |
| *Bombus* (*Pyrobombus*) *impatiens* | Illinois, USA | AY737349 | AF492875 | DQ788209 | AF493009 | - | [3] |
| *Bombus* (*Pyrobombus*) *perplexus* | Ontário, Canada | AY737373 | AF492878 | DQ788254 | AF493012 | - | [3] |
| *Bombus* (*Subterraneobombus*) *appositus* | Utah, USA | DQ787965 | DQ788395 | DQ788148 | DQ788314 | - | [4] |
| *Bombus* (*Subterraneobombus*) *borealis* | Maine, USA | DQ787981 | AF492909 | AF492976 | AF493043 | - | [4] |
| *Bombus* (*Thoracobombus*) *fervidus* | Missouri, USA | AY268406 | AF492863 | AF492930 | AF492997 | - | [4] |
| *Bombus* (*Thoracobombus*) *pauloensis* | Minas Gerais, Brazil | _ | KT187910 | KT187930 | _ | - | [9] |
| *Bombus* (*Thoracobombus*) *pennsylvanicus* | Missouri, USA | EF032352 | DQ788479 | EF032374 | AF492996 | - | [4] |
| *Bombus* (*Thoracobombus*) *pseudobaicalensis* | Primorskiy Kray, USA | EF032357 | AY267171 | EF032372 | AY267155 | - | [4] |
| *Bombus* (*Thoracobombus*) *schrencki* | W. Khentey, Mongolia | AF364828 | AF492866 | AF492933 | AF493000 | - | [4] |
| *Cephalotrigona capitata* | San Martín, Peru | FJ041915 | FJ042191 | FJ042292 | FJ042394 | FJ042015 | [7] |
| *Dactylurina staudingeri* | Cameroon, Yaoundé | DQ790451 | DQ813034 | DQ813111 | DQ813189 | FJ042176 | [7] |
| *Duckeola ghilianii* | Amazonas, Brazil | FJ041917 | FJ042193 | FJ042294 | FJ042396 | FJ042018 | [7] |
| *Friesella schrottkyi* | São Paulo, Brazil | FJ041922 | FJ042198 | FJ042299 | FJ042401 | FJ042023 | [7] |
| *Frieseomelitta trichocerata* | Loreto, Peru | FJ041923 | FJ042199 | FJ042300 | FJ042402 | FJ042024 | [7] |
| *Frieseomelitta varia* | São Paulo, Brazil | FJ041924 | FJ042200 | FJ042301 | FJ042403 | FJ042025 | [7] |
| *Geotrigona mombuca* | São Paulo, Brazil | FJ041928 | FJ042204 | FJ042305 | FJ042407 | FJ042029 | [7] |
| *Lestrimelitta limao* | São Paulo, Brazil | FJ041931 | FJ042207 | FJ042308 | FJ042410 | FJ042032 | [7] |
| *Leurotrigona muelleri* | São Paulo, Brazil | FJ041933 | FJ042209 | FJ042310 | FJ042412 | FJ042034 | [7] |
| *Leurotrigona pusilla* | San Martín, Peru | FJ041932 | FJ042208 | FJ042309 | FJ042411 | FJ042033 | [7] |
| *Meliplebeia becarii* | Mgahinga, Uganda | AF343109 | - | - | - | - | [87] |
| *Melipona* (*Eomelipona*) *bicolor* | Minas Gerais, Brazil | FJ041919 | FJ042195 | FJ042296 | FJ042398 | FJ041919 | [7] |
| *Melipona* (*Eomelipona*) *marginata* | São Paulo, Brazil | FJ041920 | EU163072 | EU163240 | FJ042399 | FJ042021 | [7] |
| *Melipona* (*Melikerria*) *fasciculata* | São Paulo, Brazil | FJ041946 | FJ042223 | FJ042324 | FJ042426 | FJ042048 | [7] |
| *Melipona* (*Melikerria*) *quinquefasciata* | Brazil | EU162989 | EU163074 | EU163242 | - | - | [63] |
| *Melipona* (*Melipona*) *favosa* | French Guiana | EU162961 | EU163046 | EU163214 | - | - | [63] |
| *Melipona* (*Melipona*) *mandacaia* | Brazil | EU162990 | EU163075 | EU163243 | - | - | [63] |
| *Melipona* (*Melipona*) *quadrifasciata* | Minas Gerais, Brazil | AF343100 | EU163069 | EU163237 | - | - | [91] |
| *Melipona* (*Michmelia*) *crinita* | Madre de Díos, Peru | FJ041942 | FJ042219 | FJ042320 | FJ042422 | FJ042044 | [7] |
| *Melipona* (*Michmelia*) *scutellaris* | Minas Gerais, Brazil | FJ041939 | FJ042216 | FJ042317 | FJ042419 | FJ042041 | [7] |
| *Melipona* (*Michmelia*) *seminigra* | Amazonas, Brazil | FJ041940 | FJ042217 | FJ042318 | FJ042420 | FJ042042 | [7] |
| *Meliponula bocandei* | Bwindi, Uganda | DQ790452 | DQ813052 | DQ813131 | DQ813209 | FJ042177 | [7] |
| *Mourella caerulea* | Rio Grande do Sul, Brazil | FJ041950 | FJ042227 | FJ042328 | FJ042430 | FJ042052 | [7] |
| *Nannotrigona testaceicornis* | São Paulo, Brazil | FJ041953 | FJ042230 | FJ042331 | FJ042433 | FJ042056 | [7] |
| *Oxytrigona tataira* | São Paulo, Brazil | FJ041960 | FJ042237 | FJ042338 | FJ042440 | FJ042062 | [7] |
| *Paratrigona subnuda* | Espírito Santo, Brazil | AF343105 | - | - | - | - | [91] |
| *Partamona auripennis* | San Martín, Peru | DQ790463 | DQ813056 | DQ813135 | DQ813213 | EU049746 | [7] |
| *Partamona testacea* | San Martín, Peru | FJ041961 | FJ042238 | FJ042339 | FJ042441 | FJ042063 | [7] |
| *Plebeina hildebrandti* | L. Trichardt, South Africa | DQ790455 | DQ813061 | DQ813140 | DQ813218 | FJ042180 | [7] |
| *Plebeia drotyana* | São Paulo, Brazil | Fj041974 | Fj042252 | Fj042353 | Fj042455 | Fj042078 | [7] |
| *Ptilotrigona lurida* | San Martín, Peru | FJ041980 | FJ042258 | FJ042359 | FJ042460 | FJ042085 | [7] |
| *Scaptotrigona bipunctata* | São Paulo, Brazil | FJ041987 | FJ042265 | FJ042366 | FJ042467 | FJ042092 | [7] |
| *Scaptotrigona depilis* | São Paulo, Brazil | FJ041988 | FJ042266 | FJ042367 | FJ042468 | FJ042093 | [7] |
| *Scaura latitarsis* | San Martín, Peru | FJ041991 | FJ042269 | FJ042370 | FJ042471 | FJ042096 | [7] |
| *Scaura longula* | San Martín, Peru | FJ041989 | FJ042267 | FJ042368 | FJ042469 | FJ042094 | [7] |
| *Schwarziana quadripunctata* | São Paulo, Brazil | FJ041992 | FJ042270 | FJ042371 | FJ042472 | FJ042097 | [7] |
| *Tetragona clavipes* | San Martín, Peru | FJ042010 | FJ042287 | FJ042389 | FJ042490 | FJ042117 | [7] |
| *Tetragonisca angustula* | São Paulo, Brazil | DQ790470 | DQ81307 | DQ813155 | DQ813233 | FJ042107 | [7] |
| *Trigona chanchamayoensis* | San Martín, Peru | EU049698 | EU049748 | EU049769 | EU049793 | EU049719 | [7] |
| *Trigona cilipes* | San Martín, Peru | EU049699 | EU049749 | EU049771 | EU049794 | EU049721 | [7] |
| *Trigona fuscipennis* | Limón, Costa Rica | EU049709 | EU049759 | EU049783 | EU049804 | HM750238 | [7] |
| *Trigona hyalinata* | São Paulo, Brazil | EU049718 | EU049768 | EU049792 | EU049813 | EU049742 | [7] |
| *Trigona hypogea* | San Martín, Peru | EU049714 | EU049764 | EU049788 | EU049809 | EU049738 | [7] |
| *Trigona pallens* | San Martín, Peru | EU049702 | EU049752 | EU049775 | EU049797 | EU049725 | [7] |
| *Trigona recursa* | São Paulo, Brasil | EU049715 | EU049765 | EU049789 | EU049810 | EU049739 | [7] |
| *Trigona spinipes* | São Paulo, Brasil | EU049716 | EU049766 | EU049790 | EU049811 | EU049740 | [7] |
| *Trigona truculenta* | Madre de Díos, Peru | EU049708 | EU049758 | EU049782 | EU049803 | EU049732 | [7] |
| *Trigona williana* | Loreto, Peru | EU049713 | EU049763 | EU049787 | EU049808 | EU049737 | [7] |
| **Outgroups** |  |  |  |  |  |  | [7] |
| *Apis dorsata* | Bangalore, India | L22893 | AY267178 | AY267146 | AF091733 | FJ042186 | [7] |
| *Euglossa imperialis* | São Paulo, Brazil | AJ581085 | AY267176 | AY267144 | AY267144 | FJ042183 | [7] |
| *Eulaema boliviensis* | La Paz, Bolivia | DQ788139 | DQ788523 | DQ788307 | DQ788387 | FJ042184 | [7] |
| *Exaerete smaragdina* | - | AJ581101 | EU421585 | AJ582379 | AJ581738 | FJ042185 | [7] |

**S1 Table additional References**

1. Costa MA, Del Lama MA, Melo GA, Sheppard WS. Molecular phylogeny of the stingless bees (Apidae, Apinae, Meliponini) inferred from mitochondrial 16S rDNA sequences. Apidologie. 2003;34: 73-84.
2. Cardinal S, Straka J, Danforth BN. Comprehensive phylogeny of apid bees reveals the evolutionary origins and antiquity of cleptoparasitism. Proc Natl Acad Sci U S A. 2010;107: 1620716211.
