# Supplementary material for: The evolution of haploid chromosome numbers in Meliponini
Source: PLoS One. 2019 Oct 24;14(10):e0224463. doi: 10.1371/journal.pone.0224463 (PMC6812824; doi:10.1371/journal.pone.0224463)
Supplement: S2 Table — (DOCX) [file pone.0224463.s004.docx]

**S2 Table.** **Probabilities (in percentage) of the haploid numbers in the reconstruction of the ancestral state between the clades.**

| **Clade** |  | **Haploid numbers (*n*) probabilities (%)** | | | | | | | | |
| --- | --- | --- | --- | --- | --- | --- | --- | --- | --- | --- |
|  | **08** | **09** | **11** | **12** | **14** | **15** | **16** | **17** | **18** | **20** |
| A | 10.5 | 8 | 1.3 | 1.3 | 1.3 | 6 | 1.4 | 17 | 52 | 1.2 |
| B | 10 | 7 | 1.3 | 1.3 | 1.3 | 6 | 1.9 | 17 | 52 | 1.2 |
| C | 10.5 | 8 | 1.3 | 1.3 | 1.3 | 6 | 1.3 | 17 | 52 | 1.2 |
| D | 9 | 20 | 1 | 1 | 1 | 5 | 1 | 45 | 16 | 1 |
| E | 9 | 20 | 1 | 1 | 1 | 5 | 1 | 45 | 16 | 1 |
| F | 0 | 100 | 0 | 0 | 0 | 0 | 0 | 0 | 0 | 0 |
| G | 0 | 0 | 0 | 0 | 0 | 0 | 0 | 0 | 100 | 0 |
